# The Impact of the Absence of Aliphatic Glucosinolates on Insect Herbivory in Arabidopsis

**DOI:** 10.1371/journal.pone.0002068

**Published:** 2008-04-30

**Authors:** Jules Beekwilder, Wessel van Leeuwen, Nicole M. van Dam, Monica Bertossi, Valentina Grandi, Luca Mizzi, Mikhail Soloviev, Laszlo Szabados, Jos W. Molthoff, Bert Schipper, Hans Verbocht, Ric C. H. de Vos, Piero Morandini, Mark G. M. Aarts, Arnaud Bovy

**Affiliations:** 1 Plant Research International, Wageningen, The Netherlands; 2 Laboratory of Genetics, Wageningen University, Wageningen, The Netherlands; 3 Netherlands Institute of Ecology (NIOO-KNAW), Heteren, The Netherlands; 4 Department of Biology, University of Milan, CNR Biophysics Institute, Milano, Italy; 5 Department of Biomolecular Sciences and Biotechnology, University of Milan, CNR Biophysics Institute, Milano, Italy; 6 School of Biological Sciences, Royal Holloway University of London, Egham, United Kingdom; 7 Biological Research Center, Szeged, Hungary; Purdue University, United States of America

## Abstract

Aliphatic glucosinolates are compounds which occur in high concentrations in Arabidopsis thaliana and other Brassicaceae species. They are important for the resistance of the plant to pest insects. Previously, the biosynthesis of these compounds was shown to be regulated by transcription factors MYB28 and MYB29. We now show that MYB28 and MYB29 are partially redundant, but in the absence of both, the synthesis of all aliphatic glucosinolates is blocked. Untargeted and targeted biochemical analyses of leaf metabolites showed that differences between single and double knock-out mutants and wild type plants were restricted to glucosinolates. Biosynthesis of long-chain aliphatic glucosinolates was blocked by the *myb28* mutation, while short-chain aliphatic glucosinolates were reduced by about 50% in both the *myb28* and the *myb29* single mutants. Most remarkably, all aliphatic glucosinolates were completely absent in the double mutant. Expression of glucosinolate biosynthetic genes was slightly but significantly reduced by the single *myb* mutations, while the double mutation resulted in a drastic decrease in expression of these genes. Since the *myb28myb29* double mutant is the first Arabidopsis genotype without any aliphatic glucosinolates, we used it to establish the relevance of aliphatic glucosinolate biosynthesis to herbivory by larvae of the lepidopteran insect *Mamestra brassicae*. Plant damage correlated inversely to the levels of aliphatic glucosinolates observed in those plants: Larval weight gain was 2.6 fold higher on the double *myb28myb29* mutant completely lacking aliphatic glucosinolates and 1.8 higher on the single mutants with intermediate levels of aliphatic glucosinolates compared to wild type plants.

## Introduction

Plants resist insect herbivory by producing a wide variety of toxic and deterrent chemicals. In Arabidopsis thaliana (Arabidopsis) and other Crucifer species, the chemical defense arsenal against insect herbivores comprises glucosinolates, alongside with protease inhibitors, phenolics and terpenoid volatiles [1], [2].

**Figure 1:**
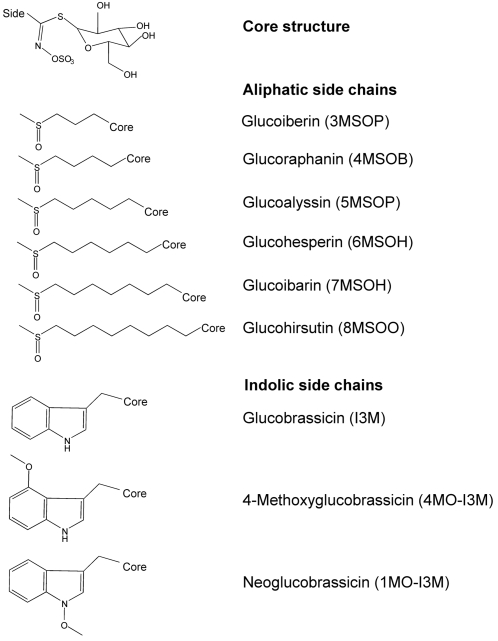
Chemical structures of the major glucosinolates in Arabidopsis thaliana Col-0.

Glucosinolates constitute a large family of secondary metabolites with over 120 different chemical structures known [3]. All glucosinolates have a core structure, composed of a β-thioglucose and an N-hydroxyiminosulphate group (Fig. 1), and an aglycone side-chain, which is structurally highly diverse. Upon tissue disruption (e.g. during herbivory), glucosinolates (which are stored in the plant vacuole) are mixed with myrosinase, a glucosidase that is spatially separated from its substrate [4]. The myrosinase activates the glucosinolates by removal of the glucose moiety. This results in the production of nitriles and (iso)thiocyanates, that are toxic and deterrent to generalist insect herbivores. A number of studies have indicated that Arabidopsis lines with high glucosinolate content show a delayed larval development of lepidopteran insects [5], [6]. Aliphatic glucosinolates may even reduce survival and growth of insects specialized in feeding on Crucifers [5].

In Arabidopsis, 36 different glucosinolates have been identified, mostly with aliphatic or indolic side-chains [7], [8]. The indolic glucosinolates are derived from tryptophane, while aliphatic glucosinolates are derived from methionine. Leaves of many *A. thaliana* accessions are very rich in aliphatic glucosinolates carrying a methylsulfinylalkyl side-chain, of which the alkyl group varies in length from 3 to 8 carbons (Fig. 1) [9].

Biosynthesis of glucosinolates involves a long series of enzymatic conversions [10]. The pathway to aliphatic glucosinolates comprises three phases, starting with deamination of methionine, followed by elongation of the side chain by sequential condensation reactions with acetyl-CoA, isomerization and decarboxylation, and finally synthesis of the core structure. Subsequently, side-chains may undergo secondary transformations, for instance to sulfinyl groups. Elongation reactions are carried out by methylthioalkylmalate synthases (MAM), an aconitase and an isopropylmalate dehydrogenase [11]. Subsequently, the glucosinolate core structure is synthesized, involving cytochrome P450 enzymes, a C-S lyase and a glucosyltransferase [10], [12]. Glucosinolate profiles are specific for species, accessions and tissues [9], [13].

Recent research has focussed on factors controlling (parts of) the glucosinolate pathway. The ability to selectively manipulate glucosinolate biosynthesis allows new opportunities in both applied and fundamental research. For applied purposes, one could aim at breeding crop plants with increased levels of glucoraphanin (4MSOB), a compound associated with lower risk of lung and colorectal cancer [14], or in increasing the total levels of glucosinolates in plants for application in biofumigation [15]. For increasing our understanding of the importance of glucosinolate biosynthetic pathways, regulating this pathway can allow to understand its role in the ecological interactions of the plant with insects and other life forms.

Recently two MYB transcription factors of the R2R3 sub-family (MYB28 and MYB29) have been identified to be involved in the regulation of the aliphatic glucosinolate biosynthetic pathway [16]–[18]. A knock-out mutation in the Arabidopsis *MYB28* gene leads to strongly reduced expression of aliphatic glucosinolate biosynthesis genes, and accordingly, the levels of long-chain aliphatic glucosinolates are significantly reduced in this mutant. For a knock-out mutation in the *MYB29* gene, no such effects were observed, suggesting that this gene was not essential for constitutive glucosinolate biosynthesis, but rather plays a role in methyl jasmonate induced glucosinolate biosynthesis [17], [19]. Over-expression of *MYB28* in Arabidopsis plants resulted in elevated levels of aliphatic glucosinolates and reduced weight-gain of *Spodoptera exigua* larvae feeding on these plants [16]. This suggests that the activity of the closely related MYB28 and MYB29 transcription factors is important for aliphatic glucosinolate synthesis and, consequently, insect resistance.

In this work, a double knock-out mutant of *MYB28* and *MYB29* was constructed in Arabidopsis. This mutant was compared to the wild type and single-mutant plants on the level of glucosinolates, gene-expression and resistance to herbivory by the generalist Lepidopteran insect *Mamestra brassicae*. The results allow a detailed insight in the role of MYB28 and MYB29 in the absolute regulation of aliphatic glucosinolate biosynthesis and their impact on the ecology of *Cruciferae*.

## Results

### *myb28* and *myb29* single and double knock-out lines

**Figure 2:**
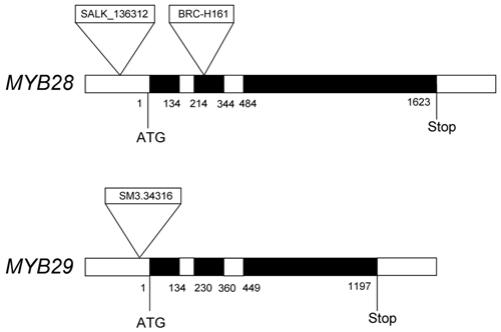
Position of the insertions in knock-out mutants of MYB28 (top) and MYB29 (bottom). Black areas represent translated regions, white areas represent untranslated regions and introns. Numbers indicate the position from the startcodon.

The function of the *MYB28* gene (At5g61420) was probed using different knock-out T-DNA insertion lines. The BRC_H161b line insertion maps in the second exon of this gene (at +242 bp from the startcodon), whereas the SALK_136312 line insertion maps 183 bp upstream of the startcodon (Fig. 2). A transposon insertion in the *MYB29* gene (At5g07690) is present in line SM3.34316. The insertion maps 44 bp upstream of the MYB29 gene startcodon (Fig. 2).

The BRC_H161b (*myb28*) line was crossed with the SM3.34316 (*myb29*) line and the progeny was self-fertilized to generate homozygous double knock-outs (*myb28myb29*). The single knock-out lines did not show any visible phenotype, whereas the double knock-out line showed a marginal delay in seed germination and initial growth. In later growth phases, there was no visible phenotypic difference between wild type Col-0 and any of the mutant lines.

### Double knock-out of *MYB28* and *MYB29* leads to complete absence of aliphatic glucosinolates

**Figure 3:**
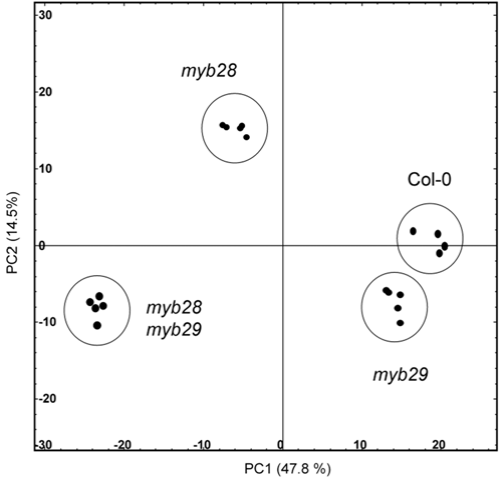
Score plot from principal component analysis of LC-MS metabolic profiles of wild type and mutant Arabidopsis lines. Contributions of principal components to separation of the samples are indicated on the axes.

The effect of the myb mutations at the biochemical level was assessed using an untargeted LC-QTOF-MS metabolic profiling approach with methanol/water extracts from mature rosette leaves. From each line, five individual replicates were analyzed. The resulting data matrix (samples vs. mass peaks) contained intensity values for 2615 mass signals (roughly representing 400 compounds) aligned across all samples. To visualize the effect of each mutation, principal components analysis (PCA) of the dataset was performed. As shown in the score plot (Fig. 3), the five biological replicates of each mutant cluster together. The plot also shows that the *myb29* mutant is relatively closely related to the wild type, while the myb28 mutant is more distinct in the plot. Remarkably, the double mutant is even more distant from the *myb28* mutant than would be anticipated from the effect of *myb29* alone.

To analyze which components lead to the separation of the mutants from the wild type, mass signals were selected that significantly differed (p<0.01; n = 5) more than two-fold in intensity between the Col-0 wild type and *myb28myb29*. In the double mutant, 159 mass signals representing 24 different compounds were found to be down-regulated: 11 compounds could be identified as glucosinolates, from the sulphinylalkyl, methylthioalkyl, phenyl and alkyl classes, while the other 13 could not be properly identified due to very low signals (<10-fold background), which do not allow accurate mass calculation and subsequent deduction of the elemental formula. In fact, in the double mutant, the identified downregulated compounds were all reduced to levels that couldn't be detected in the MS. The identified compounds are listed in Table 1. In addition, six compounds (45 mass peaks) were found to be significantly up-regulated by more than two-fold in the double mutant. Among these compounds were two indole glucosinolates (Table 1) and four unidentified compounds with very low intensity signals. Phenolic compounds such as flavonoids and sinapates, which can also affect insect resistance, were specifically assessed, but no strong changes could be observed for e.g. sinapoylmalate and kaempferol-glucoside-rhamnoside (Table 1). Apparently, the myb28 and myb29 mutations do not lead to pleiotropic phenotypes.

**Table 1:** Metabolites detected by LC-QTOF-MS (ESI negative mode) that were significantly different (student t-test, p<0.05, n = 5) between *myb28myb29* double mutant and wild-type, and relative levels of some other relevant compounds.

Retention time	Measured mass (m/z)	Calculated mass (m/z)	Elemental composition	Difference measured vs. calulated (ppm)^a^	Compound identity	Ratio double mutant / WT^b^	P-value (n = 5)^c^
3.53	422.0259	422.0255	C_11_H_21_O_10_NS_3_	1.0	glucoiberin (3MSOP)	0.00028^e^	8.7E-13
3.92	436.0414	436.0411	C_12_H_23_O_10_NS_3_	0.6	glucoraphanin (4MSOB)	0.00027^e^	2.7E-15
5.29	450.0560	450.0568	C_13_H_25_O_10_NS_3_	−1.7	glucoalyssin (5MSOP)	0.00082^e^	3.7E-09
8.80	464.0716	464.0724	C_14_H_27_O_10_NS_3_	−1.8	glucohesperin (6MSOH)	0.0056^e^	6.9E-08
13.84	478.0885	478.0881	C_15_H_24_O_10_NS_3_	0.9	glucoibarin (7MSOH)	0.00040^e^	4.1E-07
16.67	420.0460	420.0462	C_12_H_23_O_9_NS_3_	−0.5	glucoerucin (4MTB)	0.00034^e^	2.4E-06
19.56	492.1052	492.1037	C_16_H_31_O_10_NS_3_	3.0	glucohirsutin (8MSOO)	0.000030^e^	4.8E-11
23.85	422.0564	422.0585	C_15_H_21_O_9_NS_2_	−4.9	gluconasturtiin (2PE)	0.0042^e^	6.8E-09
29.37	402.0903	402.0898	C_13_H_25_O_9_NS_2_	1.3	hexylglucosinolate I^d^	0.0039^e^	6.9E-08
30.82	402.0887	402.0898	C_13_H_25_O_9_NS_2_	2.7	hexylglucosinolate II^d^	0.010^e^	4.6E-07
39.20	416.1075	416.1049	C_14_H_27_O_9_NS_2_	6.3	heptylglycosinolate^d^	0.0041^e^	4.5E-08
19.38	447.0542	447.0537	C_16_H_20_O_9_N_2_S_2_	1.0	glucobrassicin (I3M)	2.74	6.0E-08
31.66	477.0645	477.0643	C_17_H_22_O_10_N_2_S_2_	0.4	neoglucobrassicin (1MO-I3M)	2.06	8.2E-05
4.50	565.0491	565.0477	C_15_H_24_N_2_O_17_P_2_	2.4	UDP-glucose	1.36	0.0021
23.29	593.1506	593.1512	C_27_H_30_O_15_	−1.0	kaempferol-glucoside-rhamnoside	0.82	0.17
16.81	385.1152	385.1140	C_17_H_22_O_10_	3.1	sinapoyl-glucoside	1.28	0.0018
26.36	339.0711	339.0722	C_15_H_16_O_9_	−3.1	sinapoyl-malate	1.19	0.034

a accuracy of the mass measurement, as represented by the difference between the calculated and the measured accurate mass, expressed in ppm of calculated mass.

b ratio of the mean mass signal intensities of the compound in both genotypes.

c significance value in Students t-test.

d Alkylglucosinolates not identified by comparison to standard but predicted from elemental composition.

e masses not detected in the double mutant: ratios have been calculated relative to the noise level.

**Figure 4:**
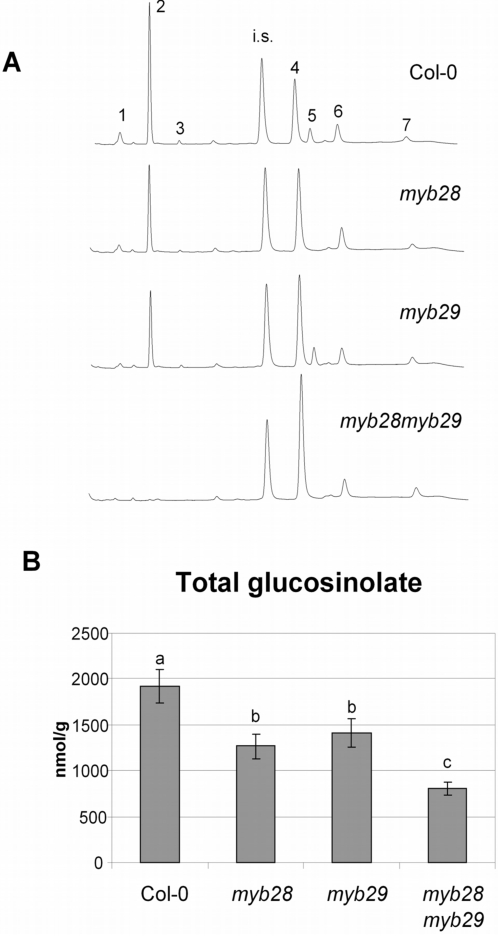
Glucosinolate contents of Arabidopsis lines. (A) HPLC profiles of glucosinolate extracts recorded at 229 nm. Numbers indicate glucosinolates: 1: glucoiberin (C3); 2: glucoraphanin (C4); 3: glucoalyssin (C5); 6: glucobrassicin (indole); 5: glucohirsutin (C8); 6: 4-methoxyglucobrassicin (indole); 7: neoglucobrassicin (indole); i.s.: internal standard (glucotropaeolin). (B) Total glucosinolate concentration in leaves of wild type (Col-0) and mutant plants (*myb28*, *myb29*, *myb28myb29*). Different letters on top of the bars indicate significance difference at p<0.05 (Tukey post hoc test).

**Figure 5 pone-0002068-g005:**
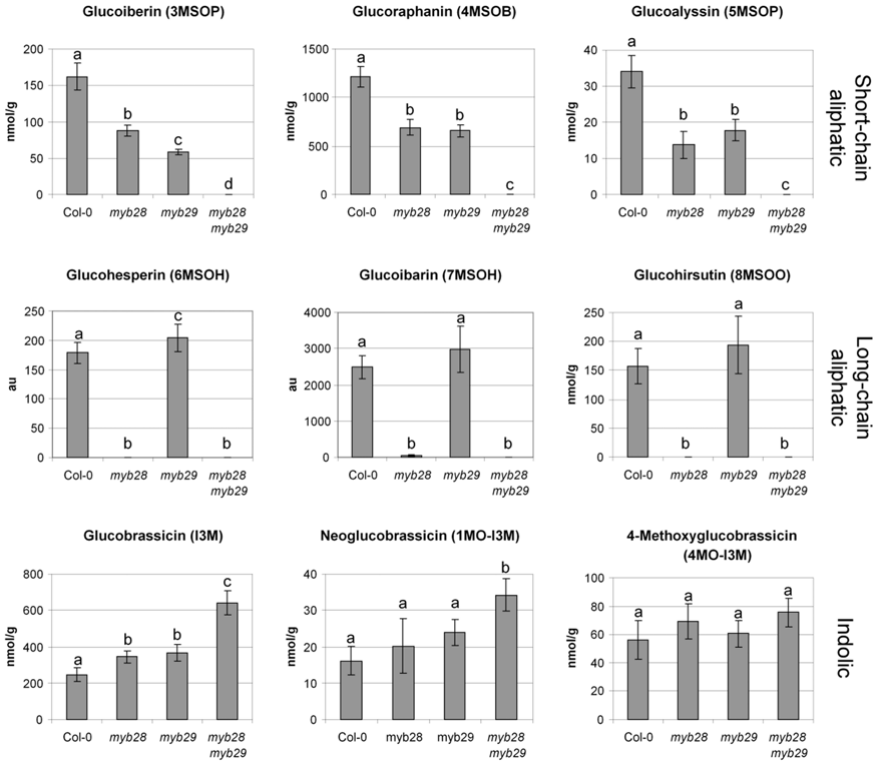
Concentration of individual glucosinolates in leaves from different Arabidopsis genotypes. Error bars indicate standard deviations (n = 5). Characters on the error bars indicate significance groups (p<0.05, Tukey post hoc test). All values were determined as nmol per g fresh weight, except for glucoibarin and glucohesperin. The latter compounds were below the detection limit in the dedicated HPLC analysis, but could be analyzed from the LC-MS analysis. Therefore they are represented as ion counts (arbitrary units; a.u.).

The glucosinolate content of the rosette leaf material was further quantified using a dedicated HPLC analysis. The HPLC chromatograms of the wild type and mutant plants are shown in Fig 4A. The total amount of glucosinolates was quantified from the chromatograms and plotted in Fig 4B. The quantification of each specific glucosinolate is shown in Fig 5. This quantitative analysis confirms the results obtained by the untargeted metabolomics analysis. Short-chain aliphatic methylsulphinylalkyl glucosinolates, such as glucoiberin (3MSOP), glucoraphanin (4MSOB) and glucoalyssin (5MSOP), are reduced by about 50% in both *myb28* and *myb29* mutants, but are completely absent from the double mutant. The long-chain aliphatic methylsulphinylalkyl glucosinolate glucohirsutin (8MSOO) is not significantly affected in *myb29*, but has completely disappeared in *myb28* and in the double mutant. Glucohesperin (6MSOH) and glucoibarin (7MSOH) showed relative changes similar to glucohirsutin (8MSOO) in the LC-MS analysis, but were below the detection level in the dedicated HPLC analysis (Fig. 5). Indolyl glucosinolates, such as glucobrassicin (I3M) and neoglucobrassicin (1MO-I3M), show a slight increase in both single mutants, and are two- to three-fold increased in the double mutant, while 4-methoxyglucobrassicin (4MO-I3M) is not significantly increased. Knock-out of both *MYB28* and *MYB29* thus completely suppressed synthesis of aliphatic glucosinolates below detection level.

### Characterization of the *myb28myb29* mutant by gene expression analysis

To further understand the mechanism by which double knock-out mutation of *MYB28* and *MYB29* genes leads to complete collapse of aliphatic glucosinolate biosynthesis, real-time RT-PCR assays were performed. Gene expression levels of *MYB28*, *MYB29* and several genes involved in aliphatic glucosinolate biosynthesis (*MAM1*, *MAM3*, *CYP83A1* and an aconitase) or indolic glucosinolate biosynthesis (*CYP83B1*) were monitored in mature expanded rosette leaf material from the Col-0 wild type, the *myb28* mutant (BRC_H161b), the *myb29* mutant, and the *myb28myb29* double mutant.

**Figure 6 pone-0002068-g006:**
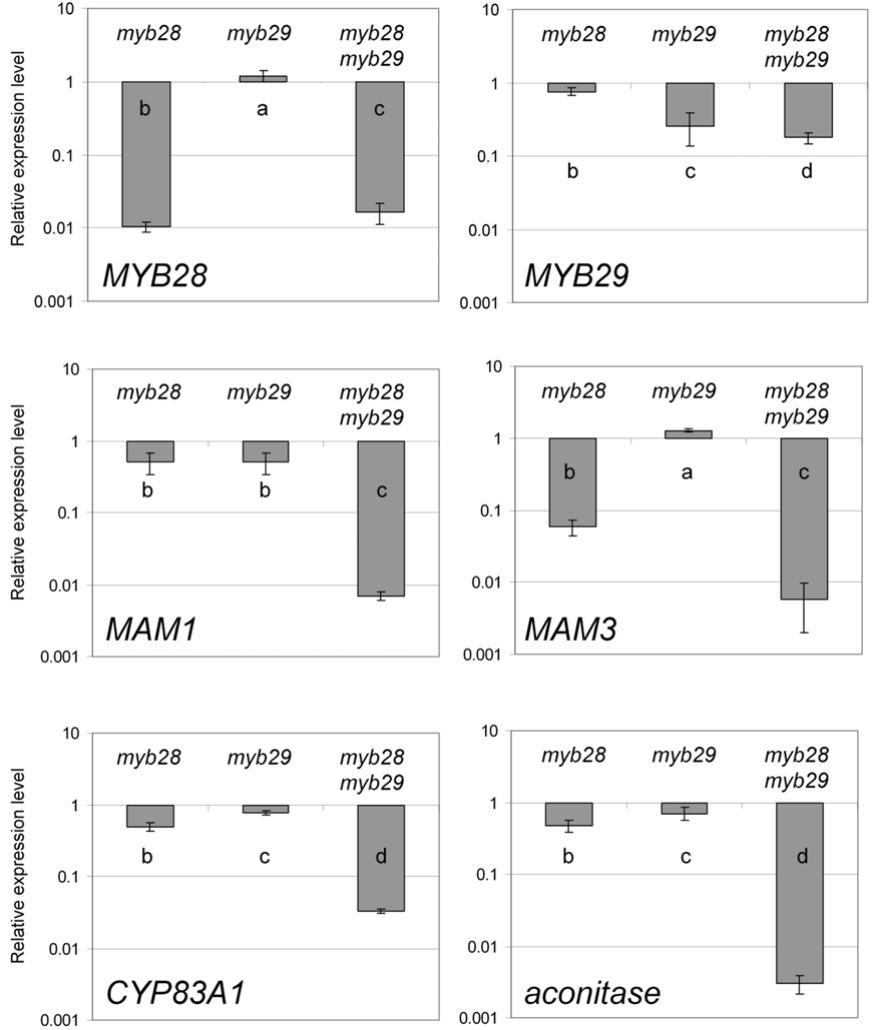
Gene expression analysis of *MYB* genes and glucosinolate biosynthesis genes. Indicated are the expression levels relative to those in the wild type on a logarithmic scale. Error bars indicate standard deviations (n = 3). Characters on the error bars indicate significance groups (p<0.05, Tukey post hoc test). The wild type values were always significance group “a”.

Compared to the wild type Col-0, the levels of *MYB28* transcripts were strongly affected (60 to 100-fold reduced) in the *myb28* and the *myb28myb29* mutants (Fig. 6). The *MYB29* transcript levels were 4 to 6-fold reduced in the *myb29* and *myb28myb29* mutant, respectively. Apparently, *myb29* is not a knock-out but a knock-down mutant, since there still is some residual expression of *MYB29* in the mutant. The *MYB28* and *MYB29* genes hardly affect each others expression in leaves.

Biosynthetic genes are dramatically more reduced in expression in the *myb29myb28* double mutant, as compared to the single mutants. Expression of *MAM3* was already strongly (>10-fold) reduced in the myb28 mutant, but even more (>100-fold) reduced in the *myb28myb29* mutant, although the expression in the *myb29* mutant was comparable to that in the wild type. The *MAM1*, *CYP83A1* and *Aconitase* transcripts were hardly affected (<2-fold) in the single mutants, but strongly reduced (140-fold, 30-fold and 300-fold, respectively) in the *myb28myb29* double mutant. On the other hand, the levels of the *CYP83B1* gene, which participates in the indolic glucosinolate pathway, were not significantly changed in any of knockout lines (data not shown). Thus, knocking out both *MYB28* and *MYB29* interfered much more severely with expression of aliphatic glucosinolate biosynthesis genes than was anticipated from the analysis of both single mutants. This suggests a strong redundancy of these transcription factors for the downstream genes tested. *MAM3* is an exception, as it is largely controlled by MYB28 and its regulation by MYB29 is epistatic to MYB28.

### Insect feeding

The *myb28myb29* double mutant is to our knowledge the first Arabidopsis genotype without aliphatic glucosinolates and it provides the first possibility to assess the relevance of aliphatic glucosinolates on herbivore insect performance. We therefore compared the performance of larvae of the lepidopteran insect *Mamestra brassicae* on the *myb28*, *myb29* and *myb28myb29* Arabidopsis mutants. *Mamestra* was chosen because its larvae are among the most frequently found pest insects on cabbages [20]. *Mamestra* is a generalist, which prefers cruciferous species, but has been found feeding on many different plant species, including non-*cruciferae* [21], [22]. There is some evidence for sensitivity of *Mamestra* to glucosinolates [23].

**Figure 7:**
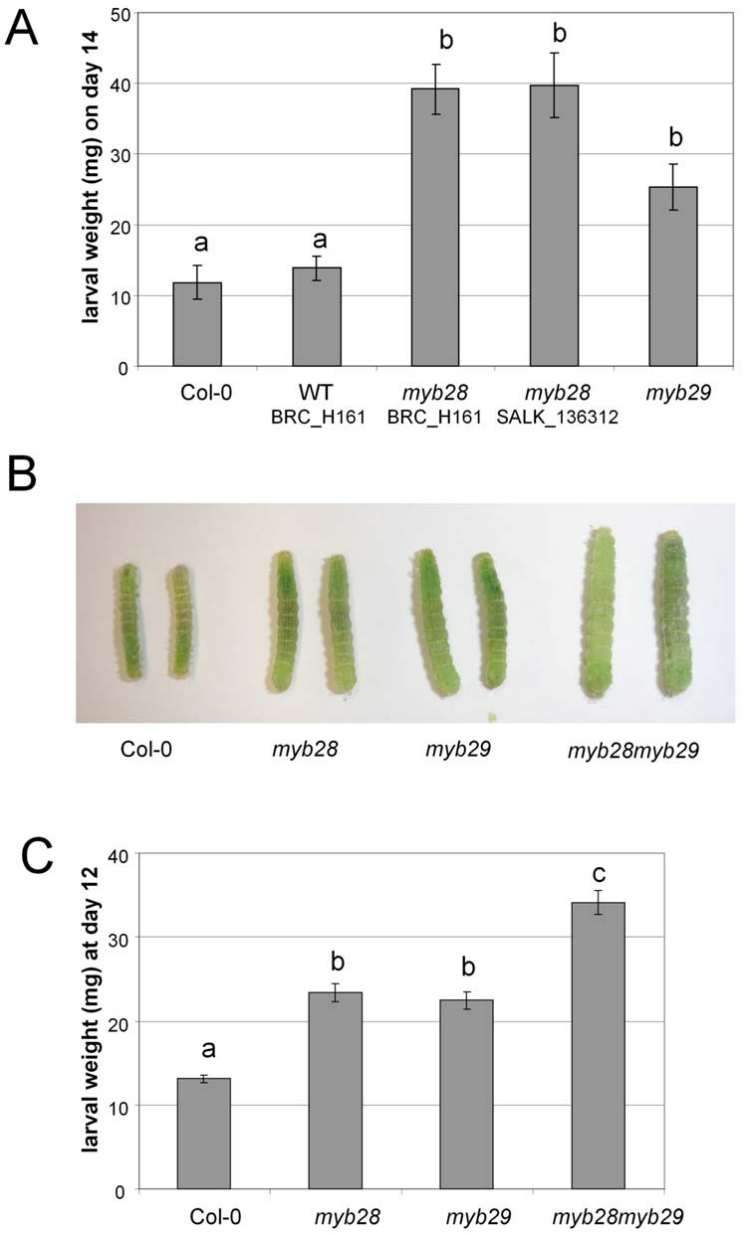
The effect of mutations in *MYB* genes on the interaction of Arabidopsis with *Mamestra brassicae*. (A) Average larval weights on day 14 of the detached leaf experiment. Error bars indicate standard errors. Different characters over the bars indicate significant differences between the treatments after Tukey's unequal N HSD analysis (p<0.05). Col-0: n = 12; WT BRC_H161: n = 15; myb28 BRC_H161: n = 17; myb28 SALK_136312: n = 17; myb29: n = 18. WT BRC_H161 is a wild type segregant obtained from the self fertilized progeny of a heterozygous *MYB28myb28* (BRC_H161) plant. myb28 BRC_H161 and myb28 SALK_H636 are homozygous *myb28* mutants carrying the BRC_H161b or the SALK_136312 T-DNA insert. *myb29* is a homozygous *myb29* mutant carrying the SM3.34316 transposable element insert. (B) Pictures of representative larvae captured from different mutant lines on day 12 of the whole-leaf experiment. (C) Average larval weights on day 12 of the whole plant experiment. Error bars indicate standard errors. Different characters over the bars indicate significant differences between the treatments after Tukey's unequal N HSD analysis (p<0.05). Col-0: n = 24; *myb28*: n = 43; *myb29*: n = 51; *myb28myb29*: n = 53.

In an initial experiment, neonate larvae were transferred to detached leaves of two wild type lines (Col-0 and progeny of a wild type segregant from a *MYB28myb28* BRC_H161b heterozygote plant), and knock-out mutants *myb28*-BRC_H161, *myb28*-SALK_136312 and *myb29*. For each experiment, individual larvae were reared separately in Petri dishes, and leaves were refreshed at least every two days. Larvae were weighed after 14 days of feeding. Mutations in *MYB28* and *MYB29* resulted in enhanced growth rates of *Mamestra* (Fig. 7A). On day 14, the average weight of larvae raised on wild type plants was two to three times lower than on leaves from knock-out plants (Fig. 7A, ANOVA F_4,76_  =  17.5, p<0.001)

In a second experiment, *Mamestra* larvae were tested on groups of hydroponically-grown intact plants. Three replicate groups of 25 neonates were confined to trays with 50 plants of wild type Col-0, *myb28*-H161, *myb29* or *myb28myb29*. After 12 days, the larvae were weighed individually. Again we found a clear effect of plant genotype on larval mass (Fig. 7B). The body mass of larvae raised on wild type Col-0 plants was significantly lower than that of larvae raised on each of the single mutants (1.7–1.8 times lower), whereas larvae on the double mutant had the highest body mass (2.6 times higher than Col-0; Fig 7C, nested ANOVA genotype effect F_3,158_  =  33.18, p<0.001). Knocking out both *MYB28* and *MYB29* genes in Arabidopsis had a significant positive effect on growth of *Mamestra* larvae, most prominently if both genes were knocked-out.

**Figure 8:**
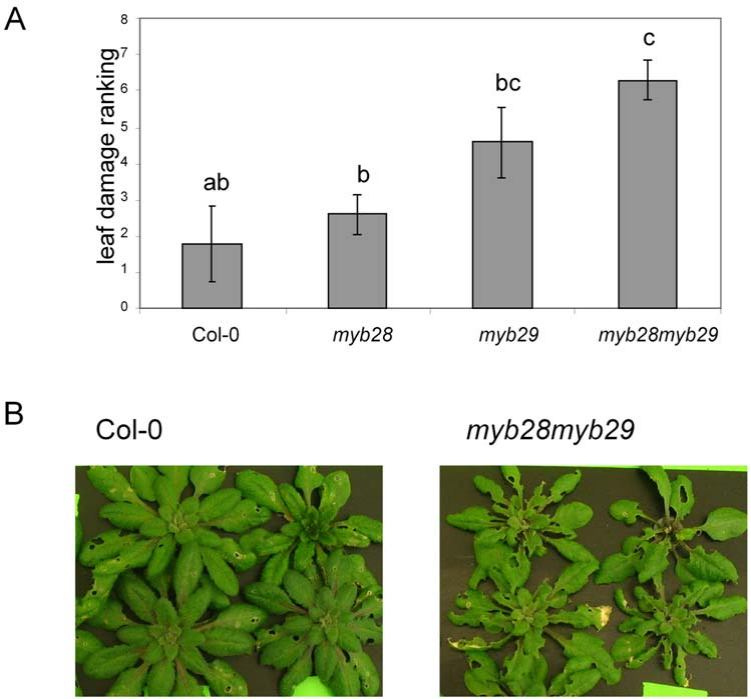
Plant damage after 10 days of *Mamestra* feeding. (A) Ranking by five observers from 1 (lowest damage) to 7 (highest damage). Different characters over the bars indicate significant differences between the treatments after Kruskal-Wallis ANOVA followed by Multiple Comparison analysis (2-tailed). (B) Pictures of representative plants of wild type Col-0 and *myb28myb29* on day 10 of *Mamestra* feeding.

In a third experiment, the effect of *Mamestra* herbivory on plants of wild type Col-0, *myb28*-H161, *myb29* or *myb28myb29* was compared. Four replicate groups of eight plants of each line were separately grown in hydroponic trays. On two of the replicates, 16 *Mamestra* neonates were positioned, while the other two replicates were not exposed to insects. After 10 days, the damage to each of the insect-treated replicates was ranked by five observers after double-blind visual inspection. As shown in Fig. 8, *Mamestra* herbivory resulted in higher damage levels in the *myb28myb29* mutant, as compared to the Col-0 wild type, while both single mutants had intermediate damage levels. These data are consistent with the higher weight of larvae feeding on the *myb28myb29* mutant.

In the same experiment, the content of glucosinolates was measured for the leaves, with and without *Mamestra* herbivory. In Table 2, the effects of herbivory for all four tested lines are shown. The values in this table were obtained by setting the concentrations of all glucosinolates in Col-0 (without herbivory) at value 1.00, and comparing the concentrations found in the other samples (with and without herbivory) to this value. In the Col-0 wild-type, three effects of *Mamestra* herbivory can be observed. Firstly, most glucosinolates (aliphatic and others) were increased by a factor 1.5 to 2. Secondly, there is a pronounced increase of the indolic glucosinolates glucobrassicin (I3M; >2 fold) and neoglucobrassicin (1MO-I3M; >6 fold). Thirdly, herbivory results in a strong decrease of glucoerucin (4MTB; a methylthioglucosinolate), which is a precursor of glucoraphanin (4MSOB; a methylsulfonylglucosinolate). In the *myb28* and *myb29* mutants, these three trends persist. For example in the *myb28* mutant, the concentration of glucoraphanin (4MSOB) is 41% of that in Col-0, and in the insect-damaged plants glucoraphanin (4MSOB) doubles to 98% of the undamaged Col-0 value (Table 2). In case of the *myb28myb29* double mutant, some traces of short-chain aliphatic glucosinolates could be observed after herbivory, up to 3% of the undamaged Col-0 levels. In this mutant, long-chain aliphatic glucosinolates such as glucohirsutin (8MSOO) could not be detected, even after herbivory, while the herbivory-induced increase in indolic glucosinolates was very pronounced (5–10 fold). These observations, made with LC-MS analysis, were confirmed by a targeted HPLC analysis (data not shown). Apparently, the *myb28myb29* double mutation strongly inhibits aliphatic glucosinolate biosynthesis, even when Arabidopsis is severely damaged by *Mamestra* larvae.

**Table 2:** Relative glucosinolate concentrations upon feeding of *Mamestra brassicae* caterpillars.

Compound	Col-0	Col-0 + insect	*myb28*	*myb28* + insect	*myb29*	*myb29* + insect	*myb28myb29*	*myb28myb29* + insect
**short-chain aliphatic glucosinolates**								
glucoiberin (3MSOP)	1	1.47*	0.52	0.98**	0.32	0.51**	n.d.^a^	0.03*
glucoraphanin (4MSOB)	1	1.93**	0.41	0.98**	0.44	0.70**	n.d.	0.03**
glucoalyssin (5MSOP)	1	1.96**	0.41	1.13**	0.56	0.92**	n.d.	0.02
**long-chain aliphatic glucosinolates**								
glucohesperin (6MSOH)	1	1.61*	0.00	0.00	0.20	1.15	n.d.	n.d.
glucoibarin (7MSOH)	1	1.83*	0.07	0.37**	0.99	1.95**	n.d.	n.d.
glucohirsutin (8MSOO)	1	2.05*	0.02	0.10**	1.04	2.35**	n.d.	n.d.
**other glucosinolates**								
glucoerucin (4MTB)	1	0.13**	0.75	0.17**	0.29	0.05*	n.d.	n.d.
gluconasturtiin (2PE)	1	1.35	0.50	0.84*	0.40	0.52	n.d.	n.d.
hexylglucosinolate I	1	1.54*	0.53	0.97**	0.47	0.96**	n.d.	0.13
hexylglucosinolate II	1	1.75**	0.33	0.84**	0.60	1.18*	n.d.	n.d.
heptylglucosinolate	1	1.36**	0.41	0.57*	0.59	0.95*	n.d.	0.04
**indolic glucosinolates**								
glucobrassicin (I3M)	1	2.63**	1.19	3.41**	1.36	4.18**	1.98	4.82**
4-methoxyglucobrassicin (4MO-I3M)	1	1.05	0.90	0.87	0.83	0.84	0.61	0.56
neoglucobrassicin (1MO-I3M)	1	6.08**	0.67	6.57**	0.84	7.51**	1.88	10.07**
**non-glucosinolates**								
UDP-glucose	1	0.93	0.91	0.75**	0.89	0.72**	0.95	0.78
kaempferol-glucoside-rhamnoside	1	0.78	0.81	0.55	0.96	0.64	0.79	0.44**
sinapoyl-glucoside	1	1.62*	0.74	1.31*	1.01	1.32	1.23	1.61
sinapoyl-malate	1	1.01	0.85	0.76	0.91	0.85	0.95	0.66*

Shown are ratios of the mass signals relative to those in control Col-0. The significance (n = 4) of concentration change due to insect feeding within the same plant line is indicated.

* : p<0.05;

** : p<0.01.

a n.d.: not detectable.

## Discussion

The transcription factors MYB28 and MYB29, together with MYB76, are known to play an important role in the regulation of aliphatic glucosinolate biosynthesis [16], [17], [19]. So far this was established by ectopically (over)expressing individual transcription factors or by studying the phenotypes of individual insertion mutants or RNAi-silenced plants. Information on redundancy of these transcription factors comes from a recent study on a double mutant in which both *MYB28* and *MYB29* are disrupted, which completely lacks aliphatic glucosinolates [18]. Our results show that MYB28 and MYB29 are largely complementary and only partially redundant with respect to the regulation of aliphatic glucosinolate biosynthesis. The presence of a functional *MYB76* gene is not sufficient to compensate for the loss of MYB28 and MYB29 function, which leads to complete absence of aliphatic glucosinolates.

### Regulation of glucosinolate biosynthesis

The double mutant *myb28myb29* has, compared to the single mutants, a unique and un-anticipated feature. All aliphatic glucosinolates (long- and short-chain) drop below the detection level, even in sensitive LC-MS analyses. In the single *myb28* line, only longer aliphatic glucosinolates are absent, while shorter aliphatic glucosinolates are only reduced to maximal 40% of the wild type level (Fig. 5). The effect of the *myb29* mutation on the biochemical level involves even less compounds (Fig. 3, 4 and 5), as was also observed by others [17], [19]. Only the shorter-chain aliphatic glucosinolates are somewhat reduced (largely to the same extent as in the *myb28* mutant), but no effect on long-chain aliphatic glucosinolates is observed. These striking differences between the double mutant and the single mutants at the biochemical level parallel those observed at the level of expression of biosynthetic genes, and have also been described in a recent publication [18]. Both the single *myb* insertion mutations lead to modest reductions in expression of structural genes in the glucosinolate pathway (up to 50% reduction; Fig. 6). There is a large overlap in the activity of MYB28 and MYB29, as also observed by others [16]–[19]. However, they are not redundant. The dramatic reduction in the expression of the glucosinolate biosynthesis genes by the *myb28myb29* double mutation indicates that MYB28 and MYB29 contribute equally to activation of these genes. Thus, the gene expression and biochemical characteristics of the double mutant show us the quantitative role of MYB28 and MYB29. The sum of the concentration of MYB28 and MYB29 quantitatively determines the level of aliphatic glucosinolates, both accounting for about 50% of this level. Our results suggest a linear correlation between the total concentration of MYB28 + MYB29 on one side and the expression of biosynthetic genes and the aliphatic glucosinolate concentration on the other.

The concentration of long-chain aliphatic glucosinolates depends mostly on the expression of the *MAM3* gene. MAM3 is essential to the biosynthesis of long-chain glucosinolates [11]. A previous report describes that *MAM3* expression is not affected in a *myb28myb29* double mutant, and is therefore probably part of a different regulatory network [18]. Our results do not confirm this, and indicate that *MAM3* expression is predominantly regulated by MYB28. The single *myb28* mutant shows a strong reduction of *MAM3* expression. Possibly, the difference between our observations on *myb28* mutants (line BRC_H161b) and those of Sønderby (SALK_136312) is the result of differences in the position of the T-DNA insertion relative to the *MYB28* open reading frame (Fig. 2). We observe no reduction of *MAM3* expression in the single *myb29* mutant. Although this suggests that *MYB29* is not at all involved in regulation of *MAM3*, knocking out *MYB29* in the absence of *MYB28* expression drastically reduced *MAM3* expression to well below levels in the single myb28 mutant (Fig. 6). Apparently the action of MYB28 on *MAM3* expression is genetically epistatic: the effect on *MAM3* of the loss of *MYB29* can only be seen in the absence of *MYB28* expression. In molecular terms, *MYB29* enhances expression of *MAM3* (as also observed by [19]), but in the *myb28* knock-out mutant, it cannot sufficiently compensate for the absence of *MYB28*. In contrast, in the *myb29* knock-out mutant, *MYB28* can readily compensate for the reduction of *MYB29* with respect to *MAM3* expression.

Interestingly, the complete down-regulation of aliphatic glucosinolate biosynthesis (by knocking out both *MYB28* and *MYB29*) leads to a significant increase in the content of indolic glucosinolates (Fig. 5). Although no significant effect on expression of *CYP83B1* was observed, the increase of glucobrassicin concentration suggests cross-talk between the biosynthetic pathways for indolic and aliphatic glucosinolates [24], [25].

### Myb mutants and insect performance

The performance of Arabidopsis-eating insects has never been tested before in the absence of aliphatic glucosinolates. In Table 2, it is clear that the phenotype of *myb28myb29*, being devoid of aliphatic glucosinolates, persists under herbivory, although some traces of short-chain aliphatic glucosinolates like glucoraphanin (4MSOB) were observed in this mutant after 10 days of *Mamestra* feeding. The results shown in Fig. 7 clearly show that *Mamestra* larvae grow faster consuming *myb28myb29* Arabidopsis, and consequently the *myb28myb29* plants suffer the highest amount of leaf damage from *Mamestra* feeding (Fig. 8). Likely, MYB28 and MYB29 contribute thereby to the plants fitness.

*Mamestra* larvae appear to particularly benefit from the reduction in short-chain glucosinolates, such as glucoraphanin (4MSOB), which is the dominant glucosinolate in Arabidopsis. In Col-0 leaves, the short-chain glucosinolate glucoraphanin accumulates to about 1200 nmol g^−1^ freshweight, which is more than 60% of the total glucosinolate content (Fig. 4). This particular compound is reduced to about 700 nmol g^−1^ in both myb28 and myb29 single mutants, and completely annihilated in the double mutant. Our results do not indicate a significant contribution of long-chain aliphatic glucosinolates to resistance to *Mamestra* in Arabidopsis. Comparison of *myb28* with *myb29* mutants, which differ only in the content of these long-chain molecules, revealed no significant difference with respect to larval weight gain or leaf damage (Fig. 7 and 8). These compounds are present in low concentrations relative to glucoraphanin (4MSOB) (Fig. 5), which apparently results in a low quantitative contribution to insect resistance.

Possibly, resistance to *Mamestra* is correlated with total glucosinolate content (compare Fig. 4B to [Fig pone-0002068-g005] [Fig pone-0002068-g006] 7C), rather than with the concentration of a particular subclass. If total glucosinolate level would be a relevant parameter, one would expect the indolic glucosinolate level, which is quite substantial and significantly increased in the double mutant (Fig. 5), also to be important for insect resistance. This would be in keeping with the observation that mainly these indolic glucosinolates are increased (up to 6 fold) upon *Mamestra* herbivory (Table 2). Indeed, it has been observed that over-expression of *MYB51*, which leads specifically to higher contents of indolic glucosinolates, has a deterrent effect on larvae of *S. exigua* [24]. However, the strong increase in indolic glucosinolates in the *myb28myb29* mutant upon herbivory is not able to compensate for the absence of aliphatic glucosinolates, for which reason the *Mamestra* larvae grow much better on this mutant. This indicates that resistance to *Mamestra* in Arabidopsis is mainly mediated by aliphatic glucosinolates, and that the strong induction of indolic glucosinolates upon herbivory is not effective to deter *Mamestra*. To understand the relevance of indolic or total glucosinolate contents on Lepidopteran herbivory, mutants that interfere with biosynthesis of these compounds, in combination with the *myb28myb29* mutant, should be explored. There are currently three MYB transcription factors (MYB34, MYB51 and MYB122) implicated in the biosynthesis of indolic glucosinolates [24]. However, MYB34 is known to affect IAA biosynthesis, and therefore pleiotropic developmental effects in such plants may be anticipated [26].

A role for the third transcription factor MYB76 which may positively regulate aliphatic glucosinolate biosynthesis does not become clear from our data. A minute amount of aliphatic glucosinolates is produced in the *myb28myb29* mutant. This could be due to also to MYB76, responding to herbivory, but also to the fact that *MYB29* expression (which is known to be jasmonate-induced) is not completely abolished in this mutant. Although over-expression of *MYB76* leads to increased synthesis of all glucosinolates [18], [19], the contribution of MYB76 is probably small in Arabidopsis leaves, where mRNA levels of *MYB76* are much lower than those of *MYB28* and *MYB29* [19].

### Application of myb mutants in ecological research

We have established a strong relation between the presence of MYB28, MYB29 and feeding by generalist insect larvae. This is reflected in both the larval weight (Fig. 7A–C) and in the damage to the plant (Fig. 8). Several studies have shown that the presence or absence of genes encoding individual enzymes involved in glucosinolate biosynthesis causes variation in glucosinolate patterns and, consequently, resistance to generalist insects [9], [27], [28]. Ecologists have postulated that differences in natural selection pressures, for example the frequencies of generalist and specialist herbivores, in local Arabidopsis populations may have caused the evolution of these polymorphisms [28]. Our results indicate that natural selection may also act on the level of transcription factors such as MYB28 and MYB29, resulting in positive Darwinian selection for entire biosynthetic pathways.

The plant response required to limit insect feeding may differ depending on the insect. In the current work it becomes apparent that aliphatic glucosinolates are important for resistance to *Mamestra*, which is a model for a generalist herbivore. It is unknown how adapted species like *Pieris rapae*, for which glucosinolates may serve as feeding stimulants [2], [29], would respond to the absence of aliphatic glucosinolates. Therefore, the role of glucosinolate-regulating MYB transcription factors in ecological interactions with other insect species, plant pathogens, nematodes and predators should be further explored. Combination of *myb* mutants affecting the aliphatic and indolic glucosinolate biosynthesis will be very interesting to further establish the roles of glucosinolate classes in ecological systems in relation to insects. The Arabidopsis *myb* mutants are excellent tools to study this and other evolutionary questions in an ecological framework.

## Materials and Methods

### Plant material

All plant material was derived from *Arabidopsis thaliana* Columbia (Col-0). A *myb28* insertion-mutation (SALK_136312) was identified in the Salk Institute T-DNA insertion collection (http://signal.salk.edu/cgi-bin/tdnaexpress). Another *myb28* insertion-mutation (BRC_H161b) was identified in the BRC collection ([30]; http://www.szbk.u-szeged.hu/arabidop/mappingoftdnalines.htm). The insertion in *MYB29* (by an En/Spm transposable element) was from the John Innes collection (SM3.34316) and obtained through NASC (N121027). Populations from the stock centers were screened for homozygous insertion by PCR with allele-specific primer pairs. To obtain a double mutant, *myb28* (BRC_H161b) was crossed with the *myb29* line. In the F2 population, double homozygous knock-outs (*myb28myb29*) were identified by PCR with allele-specific primer pairs. These plants were self-crossed, and further progeny from a homozygous line was used for experiments.

### Insect feeding

Detached leaf experiment: Arabidopsis plants (Col-0 and mutants) were grown in climate rooms with an 8 h light / 16 h darkness regime (light intensity 120 µmol m^−2^ s^−1^) at 20°C in soil. From 30-day old plants, leaves were detached with a sharp razor, and gently inserted pair-wise into 0.5 ml semi-solid water with 0.5% agar in a 0.5-ml reaction tube. For each line, twenty neonates of *M. brassicae* (Cabbage moth; Laboratory of Entomology, Wageningen University) were individually combined with leaves in sealed Petri dishes with ventilation holes, kept at room temperature and under natural daylight conditions. Every second day, leaf material was refreshed. Individual insects were weighed to the nearest 0.1 mg at day 14. Larval masses were log-transformed to meet assumptions of normality and homogeneity of variance. The log-transformed data were analyzed by ANOVA, followed by Tukey unequal N HSD analyses to identify significant differences between treatment groups.

Whole-plant experiment: Seeds were sown in Petri dishes on water-saturated filter paper followed by a 4-days cold treatment at 4°C. They were then transferred to agar filled tubes and grown on hydroponics solution [31] in trays of 50 plants. Plants were grown in a growth chamber with a 12 h light period at 20°C, 70% relative humidity and a light intensity of 35 W m^−2^. After 24 days of plant growth, *Mamestra* neonates were transferred to each tray of 50 plants. Insects weight was determined individually after 12 days. The larval mass data were log-transformed and analyzed with a nested ANOVA (tray nested in genotype) and Tukey unequal N HSD analysis. For statistical analyses Statistica 7.1 (Statsoft Inc., Tusla, OK, USA) software was used. Plant damage was determined by photographing the insect-exposed trays after 10 days. Photos were visually inspected for damage by five experienced observers and double blind ranked from low (value 1) to high (value 7) damage (10 replicates: two trays per line, five observers). The differences in ranks per plant line were analyzed by non-parametric Kruskal-Wallis ANOVA.

### Untargeted biochemical analysis

Leaves from five plants per line were snap-frozen in liquid nitrogen, snap frozen and ground to a fine powder, under continuous cooling. For metabolite profiling using LC-MS, 500 mg material was extracted using 5.0 ml 0.1% formic acid (v/v) in 75% aqueous-methanol, as described before [32].

Extracts (3 µl) were subjected to a non-targeted LC-MS based metabolomics approach [33], using an Alliance HPLC system, a PDA detector and a high resolution quadrupole time-of-flight (QTOF) MS (Waters). Electrospray ionization in negative mode was used to ionize compounds separated by the reversed phase C18 column. Data were processed by extracting mass signals and aligning them across all samples in an unbiased manner using the dedicated Metalign™ software (www.metalign.nl), and a data matrix of intensities of all mass signals × samples was created. Mass signals with an intensity <10 times the local noise in all samples were filtered out. All analyses were performed using 5 biological replicates. For multivariate analysis, the LC-MS data were read into GeneMaths software (Applied Maths, Belgium) after 2log-transformation of mass signal intensities. Mass signals (variables) were normalized by dividing by the mean of each variable.

### Targeted glucosinolate extraction and quantification

Glucosinolate extraction was basically performed as described before [9], [34]. All rosette leaves of five 23-day old plants were pooled and frozen in liquid nitrogen, in five portions per plant line. The frozen leaf material was ground in a pre-cooled metal container with a 10 mm glass bead in a Braun Mikrodismembrator U for 90 sec at 2000 rpm. Subsequently, 100 mg (fresh weight) of frozen ground leaves were weighed and 50 µl of 3 mM glucotropaeolin was added as an internal standard. Glucosinolates were extracted by adding 1 ml boiling 80% methanol, vigorous vortexing and 5 minutes incubation in an 80°C heat block. Samples were centrifuged for 1 min at 16,000 g after which extraction was repeated. Supernatants were collected and glucosinolates were absorbed on diethylaminoethyl Sephadex A-25 (equilibrated with water) in 96-well filter plates (Millipore, Tempe, AZ, catalogue no. MAHVN4550). Columns were washed twice with 0.5 ml 20 mM NaAc (pH 4), after which glucosinolates were desulphated on column by addition of 75 µl of a fresh sulphatase (25 mg ml^−1^) solution and overnight incubation at room temperature. The desulphated glucosinolates were eluted using 2 times 100 µl milliQ water, and 20 µl of each sample was analyzed with a Novapack C18 column on a Spectra Physics HPLC. Compounds were detected at 229 nm after separation using a gradient from 0% to 20% acetonitrile gradient in 0.05% tetramethylammoniumchloride in water in 20 minutes at a flow of 1 ml min^−1^. Glucosinolates were identified based on comparison to reference material. Peak area was calculated and converted to nanomoles per gram fresh weight using the internal standard peak area as a reference.

### Gene expression analysis

Total RNA was isolated from 100 mg Arabidopsis leaves (3 batches per line) using 1.5 ml Trizol reagent (Invitrogen) according to the manufacturer's instructions. RNA was treated with DNaseI (Invitrogen), and subsequently repurified using RNeasy (Qiagen). RNA concentrations were determined and 1 µg RNA was used for cDNA synthesis, using the iScript cDNA synthesis kit (BioRad). Subsequently, equal amounts of each cDNA were used in triplicate for PCR amplification using iQ SYBR Green Supermix (BioRad) on a MyiQ iCycler (BioRad), with primer pairs shown in Table 3. Data were analyzed using IQ5 Optical System software (2.0; BioRad). Threshold values (Ct) were determined in the different samples. Ct values from the beta-actin primer pair were used as reference, and subtracted from the test-gene Ct values (ΔCt). In wild type Col-0 samples, Ct values for all tested genes were between 20 and 25. Relative gene expression levels, compared to Col-0, were calculated according to Livak [35]. Technical variation between gene expression levels remained below 5% within one sample.

**Table 3:** Genes and primer pairs used for quantitative RT-PCR analysis.

Gene ID	Oligo name	Sequence	Position from ATG	Length
At5g61420	MYB28 F	AGACTTCTTGGGAAACATCGG	732	241
	MYB28 R	CACTGAGCAGATTCGCAATG	973	
At5g07690	MYB29 F	CAATACTGGAGGAGGATATAACC	1166	163
	MYB29 R	AGTTCTTGTCGTCATAATCTTGG	1329	
At5g23010	MAM1 F	TTGAGGAGGTCGTGATGG	992	187
	MAM1 R	CTGATGAATGCCGCTCTC	1179	
At5g23020	MAM3 F	TCTGAAGGCATTAGTGGTGAAC	1407	95
	MAM3 R	GCGGAAATCTGAGGGCTTG	1502	
At4g13770	CYP83A1 F	ATAGTATATGTTCCTCCAGTGTATTC	1547	78
	CYP83A1 R	GAGAAAGATAGAGAGACGATTGC	1625	
At4g31500	CYP83B1 F	TAAAGGCAGTCATCAAGG	1049	178
	CYP83B1 R	CTCATTAGGGTTGTCTCC	1227	
At2g43100	Aconitase F	GTTTGTTTGATTTGGTATTGTTGTTG	864	96
	Aconitase R	ACACTCACCATATTCACATATCTTG	960	
